# Demographic and clinical baseline characteristics from the Spanish SURVIVE prospective cohort study on suicide attempts

**DOI:** 10.1192/j.eurpsy.2025.10143

**Published:** 2026-04-28

**Authors:** Victor Perez, Matilde Elices, Carlos Schmidt, Jorge Andreo-Jover, Wala Ayad-Ahmed, María Teresa Bobes-Bascarán, María Ángeles Botí, Ana Isabel Cebria Meca, Manuel Canal-Rivero, Irene Canosa, Benedicto Crespo-Facorro, Alejandro de la Torre-Luque, Marina Díaz-Marsá, Jennifer Fernández-Fernández, Ainoa García-Fernandez, Iria Grande, Luis Jiménez-Treviño, Ana González-Pinto, Diego J. Palao Vidal, Ángela Palao-Tarrero, Andres Pemau, Ivan Perez-Diez, Beatriz Orgaz, Natalia Roberto, SURVIVE Consortium, Pilar Alejandra Saiz

**Affiliations:** 1 Hospital del Mar, Spain; 2 Hospital del Mar Medical Research Institute: Institut Hospital del Mar d’Investigacions Mèdiques, Spain; 3 Hospital La Paz: Hospital Universitario La Paz, Spain; 4 Hospital Clínico San Carlos: Hospital Clinico San Carlos, Spain; 5 Universidad de Oviedo, Spain; 6 Hospital Clínic Barcelona: Hospital Clinic de Barcelona, Spain; 7 Corporació Sanitària Parc Tauli: Consorcio Corporacion Sanitaria Parc Tauli, Spain; 8 Hospital Universitario Virgen del Rocío: Hospital Universitario Virgen del Rocio, Spain; 9 Universidad Complutense de Madrid, Spain; 10 Hospital Universitario Araba, Spain; 11 Universidad Autonoma de Madrid - Campus de Cantoblanco: Universidad Autonoma de Madrid, Spain; 12 Clínic Barcelona: Hospital Clinic de Barcelona, Spain

**Keywords:** adolescents, adults, cohort study, mental health, suicide attempt

## Abstract

**Background:**

Cohort studies of individuals with a suicide attempt are crucial for identifying risk and protective factors to prevent recurrence. The SURVIVE prospective cohort study aims to investigate the demographic and clinical profiles of individuals presenting with suicidal behaviour.

**Methods:**

A total of 1,741 individuals (289 adolescents aged 12–17 and 1,443 adults aged 18 and older) were recruited from emergency departments at eight hospitals across five Spanish regions following a suicide attempt. Baseline data were collected using structured clinical interviews and validated self-report instruments. Sociodemographic and psychiatric variables were analysed.

**Results:**

Most participants were female, and approximately 20% were migrants. Religious affiliation was reported by 35.6% of adolescents and 48.4% of adults. Depression and anxiety were the most prevalent psychiatric diagnoses, while trauma-related and eating disorders were more frequent in adolescents, and substance use disorders in adults. Non-suicidal self-injury was reported by 76.6% of adolescents and 40.1% of adults. The most common method of attempt was self-poisoning in both groups. Psychotropic medication use was widespread, with both groups receiving a similar number of prescriptions.

**Conclusions:**

The SURVIVE study provides a detailed characterization of suicide attempters in Spain, highlighting age-specific clinical patterns and contextual risk factors such as migration and religion. These findings underscore the need for tailored prevention strategies that consider developmental stage, psychiatric comorbidity, and social vulnerability.

## Introduction

Suicide remains a significant global public health concern, with over 727,000 deaths reported annually, according to the World Health Organization [1]. It is currently the 15th leading cause of death worldwide and the second leading cause among individuals aged 15–29 years [2]. In Spain, recent data from the National Institute of Statistics (INE) indicate a concerning upward trend in suicide mortality, highlighting the urgent need for evidence-based prevention strategies to reduce this burden [3].

Suicidal behaviour is a complex, multifactorial phenomenon influenced by biological, psychological, social, and environmental factors [4]. Among the most critical predictors of suicide are previous attempts: it is estimated that for every completed suicide, there are approximately 25 attempts [5]. Previous attempts are among the strongest predictors of suicide: for every death by suicide, there are estimated 25 attempts [5], and approximately one in five individuals admitted to an emergency department following a suicide attempt will reattempt within a short period, emphasizing the need for targeted interventions aimed at preventing recurrence [6].

Effective suicide prevention requires robust, population-based registries that can guide the design and implementation of tailored intervention programmes. Cohort studies play a crucial role in this context, enabling researchers to identify high-risk populations and track suicidal behaviour over time [7]. Understanding the baseline characteristics of individuals who attempt suicide – and how these factors relate to the likelihood of reattempt – is essential for developing precise, evidence-informed prevention efforts [8].

Over the past two decades, several international cohorts have investigated suicidal behaviour, differing in inclusion criteria, sample size, geographic scope, follow-up duration, and data collection methods [7, 9–13]. Despite methodological variability, consistent patterns have emerged: a predominance of female participants – particularly in younger samples – high psychiatric comorbidity (notably mood, anxiety, and personality disorders), frequent prior suicide attempts, and elevated rates of alcohol misuse [7, 9, 12, 14, 15]. Self-poisoning has been identified as the most frequent method of attempted suicide across various countries and healthcare settings [16]. Participants are often unemployed, single, or living alone, and report low educational attainment or socioeconomic status [7, 15].

In Spain, no cohort has yet included participants across broad age ranges and multiple catchment areas nationwide using standardized clinical assessments. [7, 12, 14]In response to this gap, the SURVIVE cohort study was established to examine the sociodemographic and mental health factors associated with suicide attempt recurrence in a nationally representative sample. Participants aged 12 years and older who present to one of eight participating emergency departments across Spain following a suicide attempt are recruited and followed annually. The primary aims of the SURVIVE cohort are to: (1) describe the sociodemographic and clinical characteristics of individuals presenting with suicide-related behaviours in Spain; (2) collect longitudinal data to identify factors associated with suicide attempt recurrence over time; and (3) evaluate the effectiveness of targeted psychosocial interventions in reducing suicidal behaviour within 12 months of the index attempt. This article presents the baseline sociodemographic and clinical characteristics of the SURVIVE cohort participants at the time of their index suicide attempt.

## Methods

### Study design

The Suicide Prevention and Intervention Study (SURVIVE) project is a collaboration between eight hospital services providing emergency care in the cities of Barcelona (Catalonia), Madrid (Autonomous Community of Madrid), Oviedo (Principado de Asturias), Seville (Andalusia), and Vitoria (Basque Country) in Spain. Recruitment locations included general hospitals with specialized psychiatric services and a dedicated psychiatric ward. A detailed account of service provision is given in Table 1. At each participating site, the ethics committee approved the study. Informed consent was obtained from all participants before any study-related procedures were conducted.

**Table 1.** Site descriptions and catchment area coverage

Site	Code	Catchment area	Description
Hospital del Mar	S1	300,000	Hospital del Mar’s catchment area is characterized by a younger demographic profile, with a high proportion of residents aged between 15 and 49 years. This area is notable for its demographic diversity, with a significant proportion of foreign-born residents.
Hospital Clinic	S2	540,000	Hospital Clínic de Barcelona is a tertiary referral centre that provides specialized services to patients from Catalonia, particularly the Vallès Oriental and Osona areas.
Consorci Corporaciò Sanitària Parc Taulí	S3	450,000	The Consorci Corporació Sanitària Parc Taulí is the reference healthcare provider for 9 municipalities in the eastern part of the Vallès Occidental region.
Hospital Universitario La Paz	S4	528,000	Hospital Universitario La Paz is a major, high-complexity tertiary referral hospital serving residents in the northern health area of Madrid with a broad spectrum of specialties.
Hospital Clínico San Carlos	S5	371,000	Hospital Clínico San Carlos primarily serves the population within health area 6 of Madrid, providing a wide range of specialties to both adult and paediatric patients.
Hospital Universitario Central de Asturias	S6	328,154	The Hospital Universitario Central de Asturias is the primary referral hospital for Health Area IV in the Principality of Asturias, predominantly serving the municipality of Oviedo.
Hospital Universitario Araba	S7	300,000	The Hospital Universitario Araba is the primary referral hospital for the territory of Álava within the Basque Country. Its main catchment area includes the majority of Álava’s residents, as well as a specific population from the Alto Deba region for medical services.
Hospital Universitario Virgen del Rocío	S8	564,000	The Hospital Universitario Virgen del Rocío is a major tertiary referral hospital in Seville, Andalusia. Its catchment area primarily covers the population of Seville and its metropolitan area. More specifically, it serves as the main hospital for the Seville health district.

### Participants

Participants were recruited between November 2020 and March 2023. Those who agreed to participate were scheduled for the assessment within 15 days of the index suicide attempt, which was conducted at the outpatient clinics of the same facility. In some cases, participants were admitted for inpatient treatment after the emergency evaluation; these assessments were performed either during the inpatient stay or shortly after discharge, always within the 15-day window. Inclusion criteria for both samples (i.e., adolescents and adults) comprise a suicide attempt (understood as a self-injurious act committed with at least some intent to die [17]) within the last 15 days, the ability to complete questionnaires and interviews, and having given informed consent. A clinician evaluated the participants to determine eligibility based on the study’s inclusion criteria and obtained their consent to be approached by a SURVIVE research assistant.

### Measures

A structured form was used to collect participants’ information, including age, sex, educational level, employment, and marital status, number of children, country of origin, religion, pharmacological treatment, family history of suicide, substances (drugs/alcohol) used before the attempt, and the method of the attempt.

When adolescent- and adult-specific versions of questionnaires were available (e.g., Mini-International Neuropsychiatric Interview (MINI), PHQ-9), the corresponding versions were administered. To reduce assessment burden, a subset of key self-report instruments was selected for each subsample.

The presence of psychiatric disorders was determined using the MINI for adults [18] and MINI for children and adolescents (MINI-KID) [19]. Suicidality was assessed using the Columbia-Suicide Severity Rating Scale (C-SSRS) [20, 21]. Participants were assessed for suicidal ideation and behaviour throughout their lifetime and during the 15 days preceding the index attempt.

A battery of self-reported measures was also administered in both adolescents and adults, including the following: (i) The Patient Health Questionnaire (PHQ-9) [22, 23] was used to assess the severity of depressive symptoms experienced over the past 2 weeks; (ii) The Barratt Impulsiveness Scale (BIS-11) [24, 25] was used to assess impulsivity traits; (iii) The Childhood Trauma Questionnaire-Short Form (CTQ-SF) [26, 27] was used to assess the exposure to childhood maltreatment, comprising five sub-scales (emotional abuse, physical abuse, sexual abuse, emotional neglect, and physical neglect), and (iv) The EuroQoL – 5 Dimensions – 5 Levels (EQ-5D-5L) [28] was used to assess quality of life, which includes five dimensions (mobility, self-care, daily activities, pain/discomfort, and anxiety/depression). A visual analogue scale (VAS) was also used to assess overall health perception, ranging from 0 (worst imaginable health) to 100 (best imaginable health).

Furthermore, the following self-report scales were utilized exclusively in the adult sub-sample: (i) The Generalized Anxiety Disorder scale (GAD-7) [29] was used to assess anxiety symptoms; (ii) The Brief Symptom Inventory (BSI) [30] was used to evaluate a global index of psychopathological symptoms; (iii) The Acquired Capability for Suicide Scale-Fearlessness About Death (ACSS-FAD) [31] was used to measure fearlessness about death and pain tolerance; and (iv) The Reflective Functioning Questionnaire (RFQ-8) [32] was used to measure capacities for hypo-mentalizing (uncertainty about mental states) and hyper-mentalizing (certainty about mental states).

In the adolescent sub-sample, the Strengths and Difficulties Questionnaire (SDQ) [33, 34] was used to evaluate behavioural and emotional difficulties using five subscales (emotional problems, conduct problems, and hyperactivity/inattention).

### Data analyses

The sociodemographic and clinical characteristics of cohort participants were obtained using descriptive statistics. For continuous variables, the Mann–Whitney U test was applied when normality assumptions were not met. For categorical variables, Pearson’s chi-square (χ^2^) test was used. Post hoc analyses were carried out using corrected standardized residuals to identify which cells in the contingency tables significantly contributed to the overall chi-square statistic [35].

## Results

### Cohort’s sociodemographic and clinical profile

Between November 2020 and March 2023, a total of 1,730 participants were included in the cohort.

The adolescent sample comprised 289 participants recruited across seven sites (site S1 does not provide mental health care for adolescents, and therefore, did not participate in recruitment). Most sites each contributed between 13 and 19% of the adolescent sample, while two sites (S5 and S7) recruited fewer than 10% of participants. Adolescents included in the cohort were predominantly female (87.2%), with a mean age of 15 years (SD = 1.51). All participants were enrolled in secondary education, and 26.6% had repeated a grade. Migrant status was reported by 19.4, and 35.6% reported religious affiliation (see Supplemenatry Table 1).

Among adolescents, suicide-related disorders were most prevalent (81.3%), followed by depressive (56.7%) and anxiety disorders (40.5%). Trauma- and stressor-related disorders (25.3%) and eating disorders (19.7%) were also frequent. Substance-related (4.8%) and bipolar disorders (1.4%) were infrequent. Antidepressants were prescribed to 49.8%, benzodiazepines to 28.7%, antipsychotics to 26.0%, and antiepileptics to 4.8%. Participants averaged 1.15 medications (SD = 1.09, range = 0–4).

Self-reported symptoms among adolescents included a mean PHQ-9 score of 17.94 (SD = 5.59) and a BIS-11 score of 59.80 (SD = 14.44). The SDQ revealed elevated scores in emotional symptoms, conduct problems, hyperactivity, and peer difficulties, with a total difficulties score of 20.62 (SD = 5.18). High scores in emotional abuse (mean = 15.27, SD = 5.59) and emotional neglect (mean = 13.53, SD = 5.26) were reported.

The adult group included 1441 participants. Recruitment took place across eight sites. Most sites contributed between 11% and 18% of the total sample, while two sites recruited fewer than 10% of participants.

70% of the adult sample were females, with ages ranging from 18 to 93 years (mean = 41, SD = 15.58). Over half (51.9%) had completed secondary education. Employment status: 40.1% employed, 27% unemployed, and 19.6% unable to work due to disability, receiving benefits, or retirement. Approximately one-third of adults reported being single (35%), while 39.5% were married or living with a partner. The remaining participants were divorced, separated, or widowed. Additionally, 50.2% of adults reported having children. Religious affiliation and migrant status were reported by 48.4 and 22.2% respectively.

In the adult sample, suicide-related (83.1%) and depressive disorders (72.0%) were the most common. Anxiety disorders were reported by 41.6% of adults, substance-related disorders by 22.1%, and bipolar disorders by 7.4%. Antidepressants were prescribed to 70.5%, benzodiazepines to 55.1%, antipsychotics to 29.2%, and antiepileptics to 21.3%. The mean number of medications was 1.86 (SD = 1.24, range = 0–5).

Adults reported a PHQ-9 mean score of 17.49 (SD = 6.20) and a BIS-11 score of 54.57 (SD = 17.53). The EQ-5D scores reflected poor quality of life, with anxiety/depression (M = 3.28, SD = 1.28) and pain/discomfort most affected (mean = 2.40, SD = 1.20). Emotional abuse and neglect emerged with the highest scores in the childhood trauma reports.

In summary, females were more prevalent in both adolescents and adults. Adults showed higher frequencies of depressive and substance-related disorders and greater use of psychotropic medication, whereas adolescents presented higher rates of eating and trauma- and stressor-related disorders. Across self-report measures, both groups displayed comparable levels of depressive symptoms, impulsivity, and functional impairment. Tables 2 and 3 present the sociodemographic and clinical characteristics of adolescents and adults in the SURVIVE cohort.

**Table 2.** Baseline demographic characteristics by age and distribution across recruitment sites

	total n	Adolescents (12–17 years), *n*, %		total n	Adults (> = 18), n, %	
		n	%		n	%
Sex, females	289	252	87.2	1441	1007	69.83
Age, mean, (SD)	289	14.98	(1.51)	1441	40.85	(15.58)
Educational level	287			1440		
Primary education					273	18.9
Secondary education		284	92.3		748	51.87
University					420	29.13
Grade repetition (%Yes)	287	77	26.6	–	–	–
Employment status	–			1432		
Unemployed		–	–		401	27.81
Employed		–	–		577	40.01
Student		284	92.3		172	11.93
Retired or work-disabled		–	–		283	19.63
Receiving government subsidies (&% Yes)	287	0	0	539	276	19.2
Marital status	–			1440		
Single		–	–		512	35.5
Married or cohabiting partners		–	–		569	39.5
Divorced, separated, or widowed		–	–		360	25
Migrant	287	233	80.6	1440	1121	77.8
Children (% Yes)	–	0	0	1440	724	50.2
Religious affiliation (% Yes)	287	103	35.6	1440	698	48.4
Site	289			1429		
S1		–	–		172	12.0
S2		54	18.7		228	16.0
S3		38	13.1		161	11.3
S4		53	18.3		254	17.8
S5		27	9.3		171	12.0
S6		47	16.3		101	7.1
S7		24	8.3		136	9.5
S8		46	15.9		206	14.4

*Note:* SD,Standard Deviation. S1 = Hospital del Mar; S2 = Hospital Clinic; S3 = Consorci Corporaciò Sanitària Parc Taulí; S4 = Hospital Universitario La Paz; S5 = Hospital Clínico San Carlos; S6 = Hospital Universitario Central de Asturias; S7 = Hospital Universitario Araba; S8 = Hospital Universitario Virgen del Rocío.

**Table 3.** Baseline clinical characteristics by age groups

Psychiatric diagnosis	total n	Adolescents (12–17 years)		total n	Adults (> = 18)	
	286	*n*	*%*	*1433*	*n*	*%*
Depressive disorders		164	56.7		1038	72.0
Suicide-related disorders		235	81.3		1197	83.1
Anxiety disorders		117	40.5		599	41.6
Substance-related disorders		14	4.8		319	22.1
Bipolar disorders		4	1.4		106	7.4
Mood dysregulation disorder		46	15.9		–	–
Obsessive-compulsive disorder		12	4.2		68	4.7
Trauma and stressor-related disorders		73	25.3		164	11.4
Eating disorders		57	19.7		131	9.1
Psychotic disorders		8	2.8		79	5.5
Attention deficit/Hyperactivity disorder		23	8.0		–	–
Disruptive, impulse-control, and conduct disorders		21	7.3		–	–
Pharmacological treatment	289			1441		
Antidepressants		144	49.8		1017	70.5
Antiepileptics		14	4.8		307	21.3
Antipsychotics		75	26.0		421	29.2
Benzodiazepines		83	28.7		794	55.1

		*Mean*	*SD*		*Mean*	*SD*
Number of prescribed drugs	289	1.15	1.09	1441	1.86	1.24
Patient health questionnaire, PHQ–9	286	17.94	(5.59)	1408	17.49	(6.20)
Barrat impulsivity scale, BIS–11	289	59.80	(14.44)	1441	54.57	(17.53)
EuroQol 5-Dimension, 5-Level questionnaire, EQ–5D–5L	285	47.71	(21.90)	1407	46.84	(23.85)
Mobility		1.17	(0.49)		1.53	(0.91)
Self-care		1.48	(0.82)		1.52	(0.92)
Usual activities		2.39	(1.18)		2.22	(1.28)
Pain/Discomfort		2.06	(1.06)		2.40	(1.20)
Anxiety/Depression		3.20	(1.14)		3.28	(1.28)
Visual analogue scale		47.71	(21.90)		46.84	(23.85)
Childhood trauma questionnaire – short form, CTQ-SF	224			1232		
Emotional abuse		15.27	(5.59)		12.85	(6.55)
Physical abuse		8.64	(4.80)		9.41	(5.75)
Sexual abuse		8.92	(5.91)		9.38	(6.56)
Emotional negligence		13.53	(5.26)		13.28	(5.74)
Physical negligence		8.44	(3.35)		8.66	(3.98)
Strengths and difficulties questionnaire, SDQ^a^	285			–		
Emotional problem		7.07	(1.90)		–	–
Conduct problem		3.80	(2.14)		–	–
Hyperactivity		6.02	(2.01)		–	–
Peer problem		3.73	(2.23)		–	–
Prosocial		8.04	(1.88)		–	–
Total		20.62	(5.18)		–	–
Externalizing		9.82	(3.39)		–	–
Internalizing		10.8	(3.34)		–	–
Generalized anxiety disorder, GAD–7^b^	–	–	–	1407	12.27	(5.27)
Brief symptom inventory, global severity index, GSI^b^		–	–	1404	1.86	(0.81)
Acquired capability for suicide scale, ACFSS^b^		–	–	1407	18.34	(6.56)
Reflective functioning questionnaire, RFQ–8 (certainty)^b^		–	–	1407	0.69	(0.72)

*Note:* ^a^This questionnaire was administered only to the adolescent group (12–17 years). ^b^These questionnaires were administered only to the adult group (≥18 years).

### Characteristics of the index suicide attempt

Poisoning was the most common method in adolescents (81.5%), followed by cutting (10.8%), jumping (3.5%), and strangulation or suffocation (2.1%). Only 6.2% reported alcohol use before the attempt, with low rates of marijuana (4.2%) and psychoactive drug use (0.3%). A family history of suicide was reported by 22.1% of adolescent participants.

Suicidal ideation was severe, with a mean score of 3.59 (SD = 1.37) on the C-SSRS and a mean intensity score of 15.26 (SD = 6.00). The average number of lifetime suicide attempts was 2.44 (SD = 2.33). Non-suicidal self-injury (NSSI) was highly prevalent (76.6%), and preparatory behaviours were also common: 33.6% had experienced interrupted attempts, 41.6% aborted attempts, and 45.1% engaged in preparatory actions. Regarding medical consequences, 12.5% of adolescents experienced moderately severe to severe medical damage.

Among adults, self-poisoning was also the predominant method (82.2%), followed by cutting (7.0%), jumping (3.2%), and strangulation or suffocation (3.2%). Pre-attempt substance use was more common in this group: 28.4% reported alcohol use, 4.4% marijuana, and 5.2% psychoactive drugs. A family history of suicide was reported by 28.9% of adult participants. C-SSRS scores showed a slightly lower severity of suicidal ideation in adults (mean = 3.29, SD = 1.55; intensity = 13.78, SD = 7.28). Adults reported a higher mean number of previous suicide attempts (mean = 3.3, SD = 5.54). NSSI was reported by 40.1% of adults. Interrupted, aborted, and preparatory behaviours were reported by 26.7, 30.0, and 39.9% respectively. Regarding lethality, 12.8% of adults experienced moderate to severe physical harm during the index attempt Table 4.

**Table 4.** Characteristics of the index suicide attempt by age groups

	total n	Adolescents (12–17 years), n, %		total n	Adults (> = 18), n, %	
	286	n	%	1434	n	%
Method used in the index attempt						
Poisoning		233	81.5		1179	82.2
Cutting instrument		31	10.8		100	7.0
Strangulation or suffocation		6	2.1		46	3.2
Jumping from high buildings		10	3.5		46	3.2
Others		6	2.1		63	4.4
Alcohol consumption 2 hours before the attempt	286	18	6.2	1434	409	28.4
Marihuana consumption 2 hours before the attempt	286	12	4.2	1434	64	4.4
Psychoactive drug consumption 2 hours before the attempt	286	1	0.3	1434	75	5.2
Family history of suicide (Yes)	286	64	22.1	1434	417	28.9
C-SSRS (Ideation), lifetime, mean, (SD)	286			1435		
Most severe ideation, range 0–5		3.59	(1.37)		3.29	(1.55)
*Intensity items, range 0–25*		15.26	(6.00)		13.78	(7.28)
Frequency		3.27	(1.51)		2.87	(1.58)
Duration		2.64	(1.38)		2.57	(1.46)
Controllability		2.84	(1.83)		2.45	(1.99)
Deterrents		2.73	(1.85)		2.49	(1.98)
Reasons		3.78	(1.70)		3.41	(2.02)
C-SSRS (Behaviour), lifetime, mean, (SD)	286			1435		
Total number of attempts		2.44	(2.33)		3.39	5.54
NSSI behaviours, YES, n, %		219	76.6		578	40.1
Interrupted attempt, YES, n, %		96	33.6		379	26.7
Aborted attempt, Yes, n, %		119	41.6		425	30
Preparatory acts or behaviour, Yes, n, %		129	45.1		566	39.9
*Actual lethality/medical damage (physical damage)*						
No or very minor to minor		128	47.8		542	39.4
Moderate		104	38.8		657	47.8
Moderately severe to severe		36	12.5		176	12.8

Abbreviations: SD, standard deviation; C-SSRS, columbia suicide severity rating scale.

## Discussion

This study provides a comprehensive overview of the baseline characteristics of individuals presenting with a suicide attempt to emergency departments across eight hospitals in five Spanish regions. To our knowledge, it represents the largest multicentre, naturalistic cohort study on suicidal behaviour in Spain to date, and one of the few international studies that includes both adolescents and adults starting from the age of 12.

This finding is consistent with previous research [7, 10, 12] and meta-analytic data [36]showing that females, particularly adolescents and young adults, have nearly twice the risk of suicide attempts compared to males. Several hypotheses have been proposed to explain this difference, including gender-specific risk factors and differences in suicide stigma and help-seeking behaviours [36, 37]. The mean age among adults is consistent with similar findings in Spanish and international cohorts [12, 38]. Educational attainment was modest, with nearly half of the adult sample having completed secondary education, and employment rates were relatively high compared to other cohorts [7, 12]. Approximately 20% of adults were receiving state benefits, a proportion similar to Kapur’s study [7].

Religious affiliation, a variable rarely reported in previous studies, was identified in over a third of adolescents and nearly half of adults. This finding invites further exploration, as prior research (e.g., MONSUE cohort [39]) has suggested that religious affiliation may exert a protective influence against more lethal suicidal behaviours.

Migration status emerged as a key factor, with about one in five participants across both age groups identified as migrants. This is comparable to data from the prior cohort studies [10, 39] and reinforces concerns raised in the literature about increased suicide risk and reduced access to psychiatric care in migrant populations [40]. Given its potential relevance, a systematic assessment of migration status in suicide research and clinical care is warranted.

Psychiatric disorders were prevalent in both age groups, predominantly depressive and anxiety disorders, confirming trends reported in other cohorts [11, 12, 14]. Trauma- and stressor-related disorders and eating disorders were more frequent in adolescents, while substance use disorders were more common in adults. Unfortunately, we did not collect data on personality disorders – particularly borderline personality disorder – which are known to be strongly associated with suicidal behaviour in youth and adults [14].

Self-reported symptom scores, including measures of depression, impulsivity, and quality of life, were in line with prior studies [9, 11] and confirmed clinically significant psychological distress in both age groups. Like other studies [11, 41], we found a high prevalence of childhood trauma, with higher rates of moderate- severe emotional abuse in the adult sample than in the adolescent sample (68% vs. 47% respectively). Childhood trauma has been linked to suicide risk both directly and indirectly [42]. Further analyses will help clarify the specific contribution of childhood trauma to the risk of suicide reattempt in this population.

Psychotropic medication use was high, with both groups receiving, on average, between 1.5 and 1.7 drugs. Antidepressants were the most frequently prescribed class, as in the study by [10], followed by benzodiazepines and antipsychotics. Of note, very few previous cohort studies provide clear and detailed data on prescribed psychotropics, preventing comparisons across samples.

The most common method of suicide attempt was poisoning in both adolescents and adults, consistent with global patterns [16]. Alcohol and psychoactive substance use before the attempt was more common among adults. Adolescents reported higher levels of suicidal ideation severity and intensity, as assessed by the C-SSRS.

The level of medical damage associated with the index attempt showed that 12–13% of participants in both age groups experienced moderately severe to severe physical consequences, indicating a substantial subgroup at high lethality risk. This aligns with findings from [12] and underlines the importance of considering physical injury severity when assessing suicide risk and planning aftercare.

Participants also reported multiple prior suicide attempts and a high frequency of behavioural indicators of suicide risk, such as aborted, interrupted, and preparatory acts. These behaviours – more commonly reported among adolescents – highlight the chronicity and seriousness of suicidal behaviour in this population and are aligned with risk models such as the C-SSRS framework.

Nearly one in four adolescents and almost a third of adults reported a family history of suicide. This adds to existing evidence that familial and genetic vulnerabilities may contribute to suicidal behaviour and suggests the importance of routine assessment of family history during suicide risk evaluations [43].

NSSI was strikingly prevalent in adolescents (76.6%) and moderately present in adults (40.1%). NSSI is a known predictor of future suicide attempts and adverse clinical outcomes, including psychiatric hospitalization and substance use [44, 45]. Consistent with previous studies [46, 47], NSSI was more prevalent among adolescents than adults. Although adolescent self-injury may remit spontaneously in some cases [48], it often signals enduring emotion-regulation difficulties and increases the risk of adverse psychosocial outcomes later in life [49]. Given these implications, ongoing monitoring of NSSI trajectories across follow-up waves of the cohort will be critical to identify persistence, remission, and associated clinical predictors. Its high prevalence in youth underscores the need for targeted early interventions that address both suicidal and non-suicidal self-harming behaviours.

The study has limitations. It does not include biological or genetic data or account for prior health service use, which could inform our understanding of help-seeking behaviour. The inclusion criteria excluded those who presented to non-hospital emergency services, and assessments occurred up to 15 days after the index attempt, possibly limiting state-sensitive variables. This timeframe was selected to ensure recruitment of individuals in close temporal proximity to the suicidal event, while allowing sufficient time for clinical stabilization and ethical feasibility of assessment. Although this window may limit the sensitivity of some state-dependent variables, it reflects the typical course of emergency care and referral pathways in Spain, where immediate follow-up evaluations are often scheduled within the first 2 weeks post-attempt. The lack of data on personality disorders and other variables, such as mental pain [50], is also a limitation, given their relevance to suicide risk. Although the SURVIVE study is based on the follow-up of a large cohort of suicide attempters, the present report is focused on the baseline characteristics of the cohort, and thus, cross-sectional by nature [51]. Nonetheless, the study’s strengths include a large, diverse sample, comprehensive assessments via structured interviews and validated self-report tools, and broad geographical coverage.

In conclusion, the SURVIVE cohort provides a robust baseline profile of individuals at high risk of suicide, revealing distinct patterns across adolescents and adults. From a clinical perspective, these findings underscore the need for comprehensive, age-sensitive assessments that address depression, trauma, and suicide-related behaviour following a suicide attempt. Incorporating contextual factors such as migration and social support could further enhance the effectiveness of suicide prevention strategies. Notably, NSSI was highly prevalent among adolescents, highlighting the need for focused prevention strategies in this subgroup. Lethality of the attempt, past suicidal behaviours, and family history further characterize a clinically severe population. These findings lay a foundation for subsequent analyses on predictors of suicidal behaviour recurrence and inform the design of tailored, age-specific interventions.

## Supporting information

10.1192/j.eurpsy.2025.10143.sm001 Perez et al. supplementary material Perez et al. supplementary material

## Data Availability

Data are available from the authors upon reasonable request.

## Supplementary material

The supplementary material for this article can be found at http://doi.org/10.1192/j.eurpsy.2025.10143.
