# Supplementary material for: Demographic and clinical baseline characteristics from the Spanish SURVIVE prospective cohort study on suicide attempts
Source: Eur Psychiatry. 2026 Apr 28;69(1):e52. doi: 10.1192/j.eurpsy.2025.10143 (PMC13177011; doi:10.1192/j.eurpsy.2025.10143)
Supplement: Perez et al. supplementary material [file S0924933825101430sup001.docx]

Supplementary Table 1. Participant Sociodemographic Characteristics by Site and Age Group

|  | Adolescents (12-17 years), n, % | | | | | | | Analysis | | Adults (>=18), n, % | | | | | | | | Analysis | |
| --- | --- | --- | --- | --- | --- | --- | --- | --- | --- | --- | --- | --- | --- | --- | --- | --- | --- | --- | --- |
|  |  | | | | | | | χ^2^, χ^2^† | *P*-value |  | | | | | | | | χ^2^, χ^2^† | *P*-value |
| Site | S2 | S3 | S4 | S5 | S6 | S7 | S8 |  |  | S1 | S2 | S3 | S4 | S5 | S6 | S7 | S8 |  |  |
| Sex, Females n (%) | 52 (96.3) | 32 (84.2) | 46 (86.8) | 24 (88.9) | 42 (89.4) | 17 (70.8) | 39 (84.8) | 10.58 | .10 | 104 (60.5) | 147 (64.5) | 118 (73.3) | 178 (70.1) | 142 (83) | 75 (74.3) | 99 (72.8) | 135 (65.5) | 28.67 | < .001 |
| Age, Mean, (SD) | 14.87 (1.54) | 15.08 (1.54) | 14.87 (1.58) | 15.15 (1.74) | 14.98 (1.40) | 15.38 (1.24) | 14.83 (1.48) | 3.28 | .77 | 42.83 (14.74) | 40.76 (16.44) | 43.16 (15.67) | 39.97 (16.31) | 37.41 (15.70) | 44.02 (13.75) | 38.65 (15.08) | 41.24 (14.82) | 24.91 | .001 |
| Educational Level, n (%) | - | - | - | - | - | - | - |  |  |  |  |  |  |  |  |  |  | 123.91 | < .001 |
| - Primary education |  |  |  |  |  |  |  |  |  | 30 (17.4) | 36 (15.8) | 26 (16.1) | 31 (12.3) | 31 (18.1) | 20 (19.8) | 25 (18.4) | 73 (35.4) |  |  |
| - Secondary education |  |  |  |  |  |  |  |  |  | 106 (61.6) | 129 (56.6) | 107 (66.5) | 98 (38.7) | 96 (56.1) | 54 (53.5) | 55 (40.4) | 95 (46.1) |  |  |
| - University |  |  |  |  |  |  |  |  |  | 36 (20.9) | 63 (27.6) | 28 (17.4) | 124 (49) | 44 (25.7) | 27 (26.7) | 56 (41.2) | 38 (18.4) |  |  |
| Grade repetition Yes, n (%) | 5 (9.3) | 7 (18.4) | 18 (34) | 9 (33.3) | 17 (37) | 10 (43.5) | 11 (23.9) | 17.66 | .007 | - | - | - | - | - | - | - | - |  |  |
| Employment status, n (%) | - | - | - | - | - | - | - |  |  |  |  |  |  |  |  |  |  | 58.68 | < .001 |
| - Unemployed |  |  |  |  |  |  |  |  |  | 35 (20.3) | 51 (22.4) | 52 (32.5) | 78 (30.8) | 45 (26.4) | 29 (28.7) | 39 (29.1) | 71 (35.1) |  |  |
| - Employed |  |  |  |  |  |  |  |  |  | 82 (47.7) | 88 (38.6) | 54 (33.8) | 110 (43.5) | 71 (41.8) | 46 (45.5) | 57 (42.5) | 59 (29.2) |  |  |
| - Student |  |  |  |  |  |  |  |  |  | 12 (7) | 32 (14) | 23 (14.4) | 24 (9.5) | 34 (20) | 7 (6.9) | 18 (13.4) | 22 (10.9) |  |  |
| - Retired or work-disabled |  |  |  |  |  |  |  |  |  | 43 (25) | 57 (25) | 31 (19.4) | 41 (16.2) | 20 (11.8) | 19 (18.8) | 20 (14.9) | 50 (24.8) |  |  |
| Receiving government subsidies Yes, n (%) | - | - | - | - | - | - | - |  |  | 29 (53.7) | 35 (50.7) | 61 (74.4) | 43 (43.4) | 25 (42.4) | 29 (63) | 9 (22) | 44 (51.2) | 51.12 | < .001 |
| Marital status, n (%) | - | - | - | - | - | - | - |  |  |  |  |  |  |  |  |  |  | 34.15 | .004 |
| - Single |  |  |  |  |  |  |  |  |  | 65 (37.8) | 99 (43.4) | 47 (29.2) | 91 (36) | 63 (36.8) | 29 (28.7) | 52 (38.2) | 62 (30.1) |  |  |
| - Married or Cohabitating partners |  |  |  |  |  |  |  |  |  | 61 (30.2) | 81 (35.5) | 71 (44.1) | 111 (43.8) | 62 (37.5) | 36 (35.6) | 58 (42.7) | 91 (44.2) |  |  |
| - Divorced, separated or widower |  |  |  |  |  |  |  |  |  | 55 (32) | 48 (21.1) | 43 (26.7) | 51 (20.2) | 44 (25.8) | 36 (35.7) | 26 (19.1) | 53 (25.7) |  |  |
| Migrants, n (%) | 13 (24.1) | 4 (10.5) | 8 (15.1) | 8 (29.6) | 10 (21.3) | 7 (29.2) | 6 (13) | 7.86 | .24 | 25 (14.5) | 64 (32.5) | 26 (16.1) | 67 (26.5) | 54 (31.6) | 14 (13.9) | 39 (28.7) | 19 (9.2) | 61.85 | < .001 |
| Children Yes, n (%) | - | - | - | - | - | - | - |  |  | 94 (54.7) | 91 (39.9) | 100 (62.1) | 129 (51) | 63 (36.8) | 53 (52.5) | 68 (50) | 119 (57.8) | 37.37 | < .001 |
| Religious affiliation Yes, n (%) | 17 (31.5) | 6 (15.8) | 41 (78.8) | 4 (14.8) | 11 (24.4) | 5 (22.7) | 19 (41.3) | 65.72 | < .001 | 43 (25) | 96 (42.3) | 46 (28.6) | 189 (75) | 87 (50.9) | 60 (60) | 40 (29.6) | 129 (62.6) | 190.38 | < .001 |

Note. χ²† = Kruskall-Wallis; S1 = Hospital del Mar; S2 = Hospital Clinic; S3 = Consorci Corporaciò Sanitària Parc Taulí; S4 = Hospital Universitario La Paz; S5 = Hospital Clínico San Carlos; S6 = Hospital Universitario Central de Asturias; S7 = Hospital Universitario Araba; S8 = Hospital Universitario Virgen del Rocío.

Significant differences were found among adult participants across recruitment hospitals. Post hoc analyses revealed the following: Regarding sex, hospital S5 recruited a higher percentage of female participants (83%) compared to other recruitment hospitals, which ranged from 60.5% to 74.3%. In terms of age, participants recruited by hospital S5 were younger (M = 37.41) compared to those recruited by hospitals S1 (M = 42.83), S3 (M = 43.16), and S6 (M = 44.02). Regarding education level, hospital S3 reported a higher percentage of participants with secondary education (66.5%). Recruitment hospital S4 and S7 reported a higher percentage of participants with university education (49% and 41%, respectively), while recruitment hospital S8 recruited more participants with primary education (35.4%). Regarding employment status, recruitment hospital S8 reported a significantly lower percentage of employed participants (29.2%) compared to other hospitals (ranging from 33.8% to 47.7%), and recruitment hospital S5 reported a higher percentage of students (20%) compared to other hospitals (ranging from 7% to 14.4%). Regarding the receipt of government subsidies, participants from recruitment hospital S7 reported significantly lower subsidy receipt (22%), while those from hospital S3 reported significantly higher subsidy receipt (74.4%) than participants from other hospitals. Regarding marital status, we found no significant post hoc differences between the categories after adjusting for the p-value. Regarding migrant participants, hospitals S2 and S5 recruited more migrants (32.5% and 31.6%, respectively), while hospital S8 recruited the lowest percentage (9.2%). Regarding participants with children, those from hospitals S2 and S5 reported lower percentages (39.1% and 36.8%, respectively), while those from hospital S3 reported significantly higher percentages (62.1%). Lastly, regarding religious affiliation, participants from hospitals S1, S3, and S7 reported substantially lower levels of religious affiliation (25.9%, 29.5%, and 30.3%, respectively). In comparison, participants from hospitals S4 and S8 reported significantly higher levels of religious affiliation (75.9% and 64.5%, respectively).
